# Gender Disparity in the Relationship between Prevalence of Thyroid Nodules and Metabolic Syndrome Components: The SHDC-CDPC Community-Based Study

**DOI:** 10.1155/2017/8481049

**Published:** 2017-05-21

**Authors:** Xiaoying Ding, Ying Xu, Yufan Wang, Xiaohua Li, Chunhua Lu, Jing Su, Yuting Chen, Yuhang Ma, Yanhua Yin, Yong Wu, Yaqiong Jin, Lihua Yu, Junyi Jiang, Naisi Zhao, Qingwu Yan, Andrew S. Greenberg, Haiyan Sun, Mingyu Gu, Li Zhao, Yunhong Huang, Yijie Wu, Chunxian Qian, Yongde Peng

**Affiliations:** ^1^Department of Endocrinology and Metabolism, Shanghai General Hospital, Shanghai Jiao Tong University School of Medicine, Shanghai 200080, China; ^2^Jean Mayer USDA Human Nutrition Research Center on Aging, Tufts University, Boston, MA 02111, USA; ^3^Department of Internal Medicine, Sijing Hospital, Shanghai 201601, China; ^4^Department of Chronic Disease Prevention and Control, Sijing Community Health Service Centre of Songjiang District, Shanghai 201601, China; ^5^Shanghai Pudong New Area Center for Disease Control and Prevention, Shanghai 200136, China; ^6^Department of Public Health and Community Medicine, Tufts University School of Medicine, Boston, MA 02111, USA

## Abstract

The study is aimed to investigate the pathogenesis underlying the increased prevalence of thyroid nodule (TN) in different levels of metabolic syndrome (MetS) components and analyze the relationships between TN and MetS components. A total of 6,798 subjects, including 2201 patients with TN, were enrolled in this study. Anthropometric, biochemical, thyroid ultrasonographic, and other metabolic parameters were all measured. There was obviously sexual difference in the prevalence of TN (males 26.0%, females 38.5%, resp.). The prevalence of TN in hyperuricemia (45.7% versus 37.4%,* P* = 0.001), NAFLD (41.2% versus 36.4%,* P* < 0.05), and MetS (41.4% versus 35.4%,* P* < 0.001) groups was significantly increased only in females. Insulin resistance [OR = 1.31 (1.15, 1.49)], MetS [OR = 1.18 (1.03, 1.35)], and diabetes [OR = 1.25 (1.06, 1.48)] were all independent risk factors for TN in total subjects, whereas, after stratified analysis of gender, MetS [OR = 1.29, (1.09, 1.53)] and diabetes [OR = 1.47, (1.17, 1.84)] are still strongly and independently associated with the higher risks of TN in female subjects, but not in males. Our results suggest that the components of MetS might associate with the higher risks of TN in women than in men, but further cohort study of this gender disparity in the association between TN and MetS is required.

## 1. Introduction

Thyroid nodule (TN), one of the most common clinical thyroid diseases, has been becoming increasingly prevalent all over the world in the last decades and its associated risk factors have received much attention [1]. It is estimated that TN affects 4% to 7% of adults by palpation and 19% to 67% with ultrasonography [2], with 5 to 10% being malignant worldwide [3, 4]. Thus, more thyroid nodule diagnoses mean more possibilities of the thyroid cancer occurrence in the future. Further study of the relevant risk factors of the TN is required.

Previous studies have showed that impaired glucose metabolism is an independent risk factor for increased thyroid volume and nodule prevalence [5–7]. Obesity was associated with higher risks of TN and thyroid cancer [8–10]. Insulin resistance (IR) was also shown to promote the formation and growth of TN [11]. Recently, it has been suggested that metabolic syndrome (MetS) was associated with the functional and morphological alterations of the thyroid gland and may be involved in the pathogenesis of TN [12, 13]. Although the metabolic risk factors such as obesity, insulin resistance, and abnormal glucose metabolism are involved in the pathogenesis of TN in patients and these have been targeted for therapeutic intervention [14, 15], however, up to now, the metabolic mechanisms facilitating TN in individuals still have not been fully investigated, but also there has been scarce literature investigating the different levels of MetS risk factors in subjects with or without TN. Little is known about the relationships between TN and the components of MetS components [16], which limits the understanding of the mechanisms of the relative crosstalk between TN and MetS. TN are most frequently observed in females and in the elderly [17, 18]; nevertheless, there is very little epidemiological data related to the gender disparity in the relationship between TN and the components of MetS in aged populations.

Based on this issue, the main purpose of this study was to investigate the prevalence of TN among a population aged over 45 years with different glucose metabolic status and to comprehensively investigate the association between TN diagnosed on ultrasonography and the MetS components in the SHDC-CDPC Community-based Study (Shenkang Hospital Development Center for Chronic Disease Prevention and Control project, Shanghai, China). A total of 7,920 individuals with age above 45 years were enrolled in the epidemiological investigation in a rural Chinese population. The different levels of metabolic indices between the TN group and control group were measured and compared. Our study would strengthen the associations between TN and the components of MetS and increase knowledge in gender disparity on the prevalence of TN.

## 2. Subjects and Methods

### 2.1. Participants and Data Collection

From October 2014 to July 2015, a total of 7,920 local inhabitants aged 45 years or older who had been living in Sijing, Shanghai, for 1 year or longer before the enrollment and represented ten rural communities, were enrolled in this cross-section survey. A comprehensive survey was administered by the trained research staff to obtain a detailed questionnaire, anthropometry index, medical history, family histories of chronic diseases, and current medication use. Meanwhile, smoking and drinking status were also recorded. Through multiple screenings, 476 individuals were excluded from the study with missing data on questionnaire, anthropometry index, demographic variables, physical examination data, or the glucose metabolic indexes. Furthermore, subjects who met the exclusion criteria, including illnesses, such as hypothyroidism, hyperthyroidism, chronic renal failure, excessive drinking (an alcohol intake > 140 g/week for men or >70 g/week for women), or current medication use affecting body composition, thyroid function, lipid profile, serum UA level, and glucose metabolic status, were excluded in the data analysis. In the end, a total of 6798 subjects and 2201 of them with TN were included in the final data analysis. The study protocol has been approved by the Committee on Human Research at Shanghai General Hospital, Shanghai Jiao Tong University School of Medicine. Written informed consent was obtained from each participant.

### 2.2. Anthropometric Measurement and Ultrasonography

All subjects had a physical examination in a fasting state. Blood pressure was measured in the all participants seated quietly for at least five minutes thrice consecutively and the average of three measurements was recorded. Waist circumference (WC) was measured in standing subjects, midway between the lower edge of the costal arch and the top of the iliac crest. Hip circumference (HC) was measured in standing subjects, around the widest portion of the buttocks. Body mass index (BMI) was calculated as body weight/height^2^ in kg/m^2^. Waist-to-hip ratio (WHR) was calculated as WC divided by HC. In a supine position and the hyperextended neck of all participants, ultrasound examination of the thyroid nodules, including the TN number and location, was performed and evaluated independently by the two senior experts using a B-mode high-resolution tomographic ultrasound system (Toshiba, Tokyo, Japan).

### 2.3. Biochemical Measurements and Calculation

Venous blood samples were collected from all participants in the morning after an overnight fasting for at least 10 hours. The subjects without diagnosis of diabetes underwent the oral standard 75 g glucose tolerance test (OGTT) and the previously diagnosed diabetes underwent the steamed bread meal test. Biochemical measurements, including plasma glucose concentrations, uric acid (UA), serum lipid profile containing levels of total cholesterol (TCH), low-density lipoprotein cholesterol (LDL-C), high-density lipoprotein cholesterol (HDL-C), and triglycerides (TG), were measured enzymatically using an automatic biochemistry analyzer (HITACHI 7600). Fasting plasma insulin (FINS) concentration, serum level of thyroid-stimulating hormone (TSH), free triiodothyronine (FT3) concentration, free tetraiodothyronine (FT4) concentration, and thyroid peroxidase antibody (TPOAB) concentration were measured by electrochemiluminescence analyzer (Roche Diagnostics, Basel, Switzerland). The homoeostasis model assessment for insulin resistance index (HOMA-IR) was calculated by multiplying fasting plasma insulin (mIU/l) and fasting plasma glucose (FPG) (mM) and dividing the result by 22.5. Beta cell function (HOMA-beta) was calculated as 20x fasting plasma insulin (mIU/l)/(FPG (mM) −3.5) ×100%. Glycosylated hemoglobin (HbA1c) was measured by high-performance liquid chromatography (D10; Bio-Rad Laboratories, Inc., CA).

### 2.4. Definition and Diagnostic Criteria

The MetS was defined according to the IDF criteria [19] with modification on WC cutoff point for an Asian population: the central obesity (defined as WC ≥ 90 cm for men or ≥ 80 cm for women; [20]), plus any two or more: (1) raised TG (≥1.7 mmol/l or specific treatment for this lipid abnormality); (2) reduced HDL-C (<1.03 mmol/l in men and <1.29 mmol/l in women or specific treatment for this lipid abnormality); (3) raised blood pressure (≥130/85 mmHg or treatment of previously diagnosed hypertension); (4) raised fasting plasma glucose (≥5.6 mmol/l or previously diagnosed type 2 diabetes). The diabetes and prediabetes were defined using criteria recommended by the ADA 2010 [21]. BMI ≥25 kg/m^2^ was defined as overweight or ≥30 kg/m^2^ was defined as obesity using criteria recommended by the World Health Organization [22]. Insulin resistance was evaluated using HOMA-IR of 2.8 or higher [23]. Hyperuricemia was defined as serum uric acid level of 420 umol/L or higher in men and 360 umol/L or higher in women, respectively [24]. Current smoking status (Yes or No) was defined according to smoking more than one cigarette daily for at least 6 months. Current alcohol consumption status (Yes or No) was defined according to drink more than one time of any type monthly. A thyroid nodule is a discrete lesion within the thyroid gland that is radiologically distinct from the surrounding thyroid parenchyma [25]. NAFLD was defined according to the “Diagnostic Criteria of Nonalcoholic Fatty Liver Disease by the Chinese Society of Hepatology” after exclusion of viral or autoimmune liver disease and excessive alcohol consumption [26]. NAFLD was ascertained using hepatic ultrasonography that revealed ultrasound beam attenuation, a diffusely increased echogenicity in the liver parenchyma or poor visualization of intrahepatic structures by two trained ultrasonographists [27].

### 2.5. Statistical Analysis

All statistical analyses were performed using the SAS version 9.2 (SAS Institute Inc., Cary, NC, USA). Demographic, metabolic features and other clinical parameters were described by sex using frequency (percentage) for categorical variables and median (interquartile range) for continuous variables, respectively. Additionally, we divided the participants into different subgroups with and without thyroid nodules according to the different levels of MetS components and clinical characteristics. Differences on metabolic characteristics in subjects with or without TN were evaluated using *χ*^2^ test for the categorical variables or using nonparameter Wilcoxon test for analysis for the continuous variables. To further explore whether metabolic syndrome is associated with the risk of TN, unconditional sex-stratified logistic regression models were used to estimate the adjusted odds ratios (ORs) and 95% confident intervals (CIs) of MetS components with TN prevalence. Significance tests were two-tailed, with* P* value less than 0.05 considered of statistical significance.

## 3. Results

### 3.1. General Characteristics of Subjects with or without Thyroid Nodules

Clinical characteristics of the total of 6,798 participants including 3289 males and 3509 females, with a median age of 58.8 years (52.5–66.0), stratified by gender with and without thyroid nodules, were presented in Table 1. Of the 6798 subjects, the prevalence of TN was 32.4%. The prevalence of TN in women was significantly higher (38.5%) than in men (26.0%) (*P* < 0.001). Regardless of gender, analysis of the clinical risk factors revealed that subjects with TN were significantly older and had higher levels of SBP, FPG, PPG, HbA1c, FINS, HOMA-IR, HOMA-beta, FT4, and TPOAB than subjects without TN. Furthermore, all subjects with TN had significantly lower levels of DBP, FT3, and TSH than those without TN. However, after further gender stratification, there were still significant differences of SBP, UA, FPG, PPG, FINS, HOMA-IR, and TSH between the two groups in females.

### 3.2. Stratified Analysis of Prevalence of Thyroid Nodules according to the Different Metabolic Status

To explore the association of TN and related metabolic risk factors, the subjects were classified into different subgroups according to the different levels of MetS components and clinical characteristics (Table 2). The prevalence of TN was significantly increasing in the elder subjects (*P* for trend <0.001) and insulin resistance group (31.0% in the total control group, 37.0% in the total IR group, *P* < 0.001; 24.9% in the male control group, 30.1% in the male IR group, *P* < 0.001; 37.2% in the female control group, 42.0% in the female IR group, *P* < 0.01), respectively. However, there were no significant differences in the prevalence of TN between the male and female subgroups with the different levels of body fat accumulation (divided by BMI; by WHR quartile; by waist circumference) except for higher prevalence of TN in total subjects with central obesity. The prevalence of TN with normal glucose metabolism, prediabetes, and diabetes was 29.9%, 31.4%, and 37.2%, respectively, whereas the significant differences were only in female subjects (33.3%, 37.9%, and 46.0%, resp., *P* < 0.001). To explore whether MetS and the associated other metabolic parameters contributed to the pathogenesis of TN, all subjects were further divided into different subgroups according to being with or without MetS, with or without NAFLD, and with or without hyperuricemia, respectively. The results showed that the prevalence of TN with hyperuricemia (45.7% versus 37.4%, *P* = 0.001), NAFLD (41.2% versus 36.4%, *P* < 0.05), and MetS (41.4% versus 35.4%, *P* < 0.001) groups was statistically significantly higher in females, but not in males.

### 3.3. Logistic Regression Analyses of the Associated Metabolic Risk Factors of TN in Male and Female Subjects

**Figure 1:**
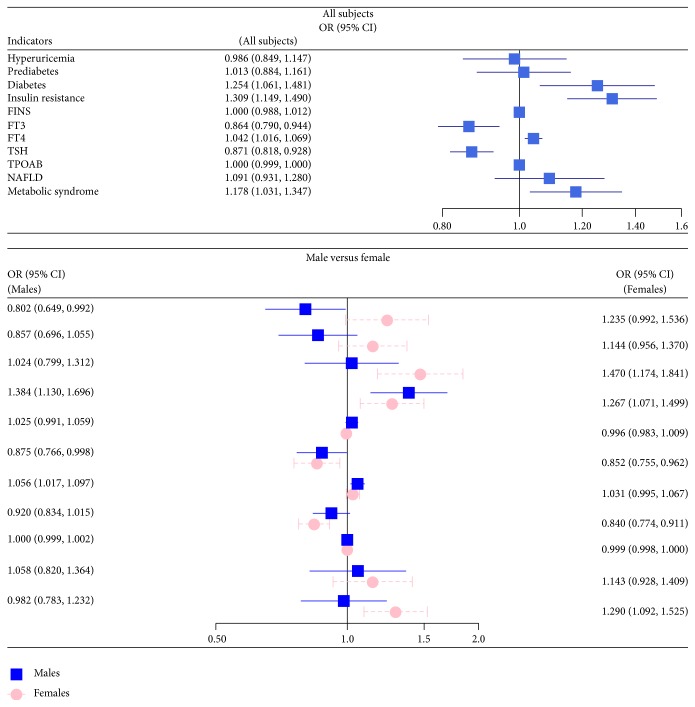
Metabolic risk factors of thyroid nodules were analyzed using logistic regression in total, male and female subjects. Adjustment of age, drinking, smoking, and family history of thyroid disease; FINS, fasting plasma insulin; FT3, free triiodothyronine; FT4, free thyroxine; TSH, thyroid-stimulating hormone; TPOAB, thyroid peroxidase antibody; NAFLD, nonalcoholic fatty liver disease.

Finally, to explore whether the MetS and the associated other metabolic parameters were independently associated with TN. A multiple logistic regression analysis for the risk factors of TN involving all the significant different anthropometric and metabolic parameters, such as central obesity, HOMA-IR, HOMA-beta, FPG, PPG, FINS, hyperuricemia, NAFLD, and MetS, were applied in subjects with or without TN (Figure 1). We next performed the stratified analysis in the subgroups divided according to the serum UA levels, glucose metabolic status, HOMA-IR, FINS, thyroid function, waist circumference, NAFLD, and MetS. Analysis of logistic regression indicated that diabetes [OR = 1.254, (1.061, 1.481)], insulin resistance [OR = 1.309, (1.149, 1.490)], and MetS [OR = 1.178, (1.031, 1.347)], but neither hyperuricemia nor FINS, were all independently significant risk factors for the increased prevalence of TN in all subjects after additional adjusting for age, smoking, drinking status, and family history of thyroid disease. Additionally, the most pronounced sex disparity was found in the relationship between the prevalence of thyroid nodules and metabolic syndrome. After stratified analysis of gender, MetS [OR = 1.29, (1.092, 1.525)] and diabetes [OR = 1.47, (1.174, 1.841)] are still strongly and independently associated with the higher risks of TN in female subjects, but not in males (Figure 1).

## 4. Discussion

The prevalence of TN and the accompanying thyroid tumors are the increasing public health problems [1, 28, 29]. Guo et al. [30] reported the population aged over 40 years had a higher prevalence (46.6%) of TN previously. Our cross-sectional study was performed in a large community-based population in rural China. Among men and women aged over 45 years, the prevalence of TN was one-third higher in women than in men (38.5% versus 26%, resp.). This frequency was higher with advancing age, among females and in subjects with insulin resistance. But, up to now, the mechanisms of the higher prevalence of TN in females as compared to the males are not completely understood. So we investigated the prevalence of TN in different levels of MetS components and examined whether MetS components and the associated metabolic risk factors contributed to the pathogenesis of TN in male and female subjects, respectively. Our results were partially comparable to previous studies, suggesting that females and the elderly were all risk factors for TN [18, 31, 32]. Moreover, we found that diabetes and MetS were independent risk factors for TN after adjusting for age, smoking, and alcohol consumption only in female subjects, but not in males.

Metabolic syndrome is a complex clinical disorder characterized by dyslipidemia, obesity, NAFLD, insulin resistance, hyperuricemia, and a disturbance of glucose metabolism. In our study, to further explore whether MetS components and the other metabolic risk parameters related to the pathogenesis of TN in subjects, all subjects were divided into different subgroups according to different metabolic status. Nearly 35.3% of subjects with MetS had TN. The results showed that the subjects with TN had significantly higher levels of FPG, PPG, HbA1c, FINS, HOMA-IR, HOMA-beta, and TPOAB than those without TN after adjusting for age. However, in addition to the above findings, after further stratified analysis of gender, there were still significant differences of FPG, PPG, FINS, HOMA-IR, and TSH between the groups with or without TN in females, but not in males. The females with hyperuricemia, NAFLD, or MetS had much higher prevalence of TN as compared with controls, whereas there were no such associations in males. Perhaps the most intriguing finding of our study was that the MetS were significantly associated with TN only in women. The one plausible explanation for this gender disparity in TN formation was the hormone testosterone, which might contribute to the protective roles against the harmful effects of MetS cluster in men compared to women. We concluded from our data that the prevalence of TN was more closely associated with the components of MetS in women than in men. Female subjects with MetS were at increased risk for TN. Few studies assessed gender disparity in the pathogenesis of TN formation [33]. The gender dichotomy of MetS-induced TN formation may underlie the increased propensity to TN in women. The gender differences observed in this study contributed to an increased theoretical understanding of TN in MetS and might suggest future studies into the sex-specific pathophysiology of TN in MetS, which remains to be further determined. Clinically, awareness of gender differences in the relationship between thyroid nodules and the components of MetS might help to detect TN in women with MetS. Therefore, better intervention strategies against the components of MetS might be performed to reduce the risks of TN occurrence.

Researchers have recently focused their interest on the pathogenesis of TN in subjects with abnormal glucose metabolism [6, 34]; however, the mechanisms facilitating TN in individuals with impaired glucose metabolism have not been investigated thoroughly [5]. In previous studies, strong correlations between thyroid volume, BMI, and WC were demonstrated [8, 16, 35–37]. Because the fat deposition in abdomen was linked to IR and MetS, the prevalence of TN in patients with central obesity or IR, which were the high risk states for development of diabetes, were significantly higher than that of controls in total subjects in our study. Ayturk et al. [38] discovered that IR was an independent risk factor for TN formation in iodine-sufficient areas, but the pathophysiologic mechanisms for the increased risk were still not fully understood. It has been reported that metformin which could improve IR might result in a significant decrease in the nodular size in patients with IR [39]. Nonetheless, there was limited data about the interaction between insulin resistance and the pathogenesis of TN [11, 40]. In this study, the prevalence of TN in diabetes group was 37.2%, which was significantly higher than 31.4% in the prediabetes and 29.9% in normal glucose tolerance (NGR). IR as an important metabolic factor in the development of the diabetes may have had an impact on the incidence of TN. We also found the risks of prevalence of TN were increased by 1.384 times in male and 1.267 times in female, with IR, respectively. It has been indicated that insulin receptors were overexpressed in most thyroid tumors. All these findings suggested the level of IR was the key factor most independently and strongly correlated with TN in all subjects. After stratified analysis of gender, MetS and diabetes were still strongly and independently associated with the higher risks of TN in female subjects, but not in males. These results indicated that there is a need for better understanding of gender disparity in the relationship between prevalence of TN and MetS components. Additional studies are required to discern how these findings may impact future research, diagnosis, and treatment of TN.

Recently, extensive studies have found the roles of NAFLD and hyperuricemia in IR. Up to now, we were unable to find any published studies to explore the role of NAFLD in pathogenesis of TN in a large population [41, 42]. The literature about the relationships between NAFLD, hyperuricemia, and TN remains scant. Concerns have been given to the relationship between hyperuricemia, inflammation, and IR recently. Since others have shown positive correlations between the levels of UA and IR [40], we inferred that the hyperuricemia may lead to the formation of thyroid nodules. The results of our study also showed that the prevalence of TN with hyperuricemia and NAFLD groups was statistically significantly higher in females. It has previously been reported that NAFLD and hyperuricemia, which by themselves are risk factors for the activation of inflammation pathways, share many predisposing metabolic risk factors with IR [43, 44]. We tentatively put forward that IR, hyperuricemia, NAFLD, and MetS might play the important roles in TN formation. Therefore, in female subjects with IR, NAFLD, hyperuricemia, or MetS, more attention should be paid to the early and the timely medical management.

Ayturk et al. reported that higher serum TSH level was an independent risk factor for increased thyroid volume in MetS patients but failed to find the relationship between TSH and TN formation [38]. In our study, regarding free thyroid hormones, results appeared contradictory at TN prevalence. Higher FT3 and serum thyroid-stimulating hormone levels were related to a decreased risk of TN in female subjects, while higher FT4 level was related to an increased risk of TN in male subjects. A recent study reported that FT3 and FT3/FT4 were positively related to BMI, waist, TG, and FPG, while FT4 was negatively related to the above metabolic parameters [45]. Few studies have explored this apparent paradox between the free THs (thyroid hormones) and TN. Taken together, although the difference of the study populations might be responsible for the above inconsistency, it was still difficult to explain the independent association between TN formation and the free THs levels. A possible explanation could be that free T3 and free T4 might be affecting the prevalence of MetS in opposite ways [45–47]. In addition, our study should be acknowledged with several potential limitations. This study was cross-sectional in design and therefore no causal inferences can be drawn. Although we found different levels of SBP, UA, HOMA-IR, FT3, and FT4 as well as plasm glucose between subjects with or without TN, it was still hard to get any causal relationships. Furthermore, the findings of single-centre study could not have been used to all TN population regarding urbanization, economic development, and geographic distribution. Given these findings, prospective studies on a larger scale are required to clarify the causal associations of MetS, IR, and hyperuricemia with TN in subjects. Therefore, the early integrated intervention involving uric acid-lowering, lipid-regulating therapy, and insulin-sensitizing medication might more effectively delay the formation of TN than either intervention alone in women with MetS.

## 5. Conclusion

Although the components of metabolic syndrome lie among the risk factors for TN both in men and women, our results suggested that MetS components had the much stronger effects on the risk of TN in women than in men. In conclusion, age, gender, IR, MetS, and abnormal glucose metabolic status as well as hyperuricemia independently played the important roles in the pathological mechanisms of thyroid nodules. The prevalence of TN in female patients with MetS was significantly increased, which was significantly associated with the different levels of MetS components. The prevalence of TN in DM group was significantly higher than those in NGR and prediabetes groups only in females. The right managements of MetS aimed to adjust hyperuricemia, central obesity, abnormal glucose metabolism, and IR might be beneficial in the blocking of TN formation, especially in females. Our data supported the possible metabolic clues to the gender disparity of nodule formation. Hence, in the future, more cross-sectional and long-term cohort multicenter study on a large scale will be necessary to further verify and clarify the findings in this study.

## Figures and Tables

**Table 1:** Clinical characteristics of the participants according to the presence or absence of thyroid nodules stratified by gender.

Parameters	Total subjects (*n* = 6798)		Statistic	*P*	Male (*n* = 3289)		Statistic	*P*	Female (*n* = 3509)		Statistic	*P*
	Thyroid nodules	Nonthyroid nodules			Thyroid nodules	Nonthyroid nodules			Thyroid nodules	Nonthyroid nodules		
Participants	2201 (32.4)	4597 (67.6)			854 (26.0)	2435 (74.0)			1352 (38.5)	2157 (61.5)		

*General indices*												
Age (years)	60.8 (55.1,67.9)	57.9 (51.4,64.9)	11.911	<0.001	61.1 (55.6,67.7)	58.1 (52,65)	8.12	<0.001	60.6 (54.8,68)	57.6 (50.7,64.8)	9.09	<0.001
Smoking, *n*, (%)	470 (22.1)	1342 (30.2)	48.20	<0.001	465 (55.4)	1332 (56.2)	0.170	0.680	5 (0.4)	10 (0.5)	0.17	0.684
Drinking, *n* (%)	226 (10.7)	802 (18.2)	60.32	<0.001	216 (26.3)	785 (33.6)	14.97	<0.001	10 (0.8)	17 (0.8)	0.02	0.883
SBP (mmHg)	136.0 (124.7,149.0)	134.0 (122.7,146.3)	4.43	<0.001	135.3 (123.7,148.3)	134.0 (123.3,146.3)	1.71	0.088	136.7 (125.3,149)	133.7 (122.3,146.3)	4.29	<0.001
DBP (mmHg)	77.0 (70.7,83.3)	77.7 (71.0,84.3)	−2.82	0.005	78.0 (71.3,84.3)	79 (72,86)	−2.81	0.005	76.7 (70.3,82.7)	76.3 (70,82.7)	0.257	0.797
*Metabolic indices*												
BMI (kg/m^2^)	24.2 (22.1,26.5)	24.3 (22.1,26.5)	−0.66	0.512	24.4 (22.4,26.5)	24.6 (22.3,26.6)	−0.42	0.672	24.1 (21.9,26.5)	24 (21.9,26.3)	0.23	0.822
WC (cm)	86 (80,92)	86 (80,93)	−0.58	0.564	88 (82,93)	87 (81,94)	0.93	0.354	84 (78,91)	84 (78,91)	0.66	0.506
WHR	0.9 (0.9,1)	0.9 (0.9,0.9)	−0.51	0.611	0.9 (0.9,1.0)	0.9 (0.9,1.0)	1.29	0.199	0.9 (0.9,0.9)	0.9 (0.8,0.9)	0.78	0.437
TCH (mmol/l)	5.1 (4.5,5.8)	5.1 (4.5,5.7)	1.90	0.057	4.8 (4.3,5.4)	4.9 (4.4,5.5)	−2.15	0.032	5.3 (4.7,5.9)	5.2 (4.7,5.9)	1.57	0.117
TG (mmol/l)	1.4 (1.0,1.9)	1.3 (0.9,2.0)	0.67	0.500	1.4 (1.0,2.0)	1.4 (0.9,2.1)	−0.12	0.902	1.4 (1,1.9)	1.3 (0.9,1.9)	1.64	0.102
HDL-C (mmol/l)	1.5 (1.3,1.8)	1.5 (1.3,1.8)	0.75	0.451	1.4 (1.2,1.6)	1.4 (1.2,1.7)	−2.95	0.003	1.6 (1.4,1.9)	1.6 (1.4,1.9)	0.03	0.973
LDL-C (mmol/l)	2.9 (2.4,3.4)	2.8 (2.4,3.3)	1.65	0.099	2.7 (2.2,3.2)	2.7 (2.3,3.2)	−1.510	0.131	3 (2.5,3.5)	2.9 (2.5,3.5)	1.24	0.215
UA (*μ*mol/l)	306 (256,363)	313 (261,369)	−2.73	0.006	353 (304,404)	350 (302,404)	0.09	0.930	280 (237,329)	273 (235,318)	3.20	0.001
FPG (mmol/l)	5.7 (5.3,6.3)	5.7 (5.3,6.2)	2.48	0.013	5.7 (5.3,6.3)	5.7 (5.3,6.3)	−0.08	0.938	5.7 (5.4,6.3)	5.7 (5.3,6.1)	4.01	<0.001
PPG (mmol/l)	7.6 (6.2,10.0)	7.3 (6.0,9.4)	5.07	<0.001	7.3 (5.9,10.1)	7.2 (5.8,9.6)	1.49	0.135	7.8 (6.5,9.9)	7.3 (6.1,9.2)	4.96	<0.001
HbA1c (%)	5.6 (5.3,6.0)	5.6 (5.3,5.9)	4.76	<0.001	5.6 (5.3,6)	5.6 (5.3,5.9)	2.26	0.024	5.6 (5.4,5.9)	5.6 (5.3,5.8)	4.71	<0.001
FINS (mIU/l)	7.2 (4.9,10.7)	6.8 (4.6,9.9)	4.10	<0.001	6.4 (4.2,9.6)	6.2 (4.1,9.2)	1.21	0.225	7.7 (5.3,11)	7.5 (5.2,10.6)	2.15	0.032
HOMA-IR	1.9 (1.2,2.9)	1.8 (1.1,2.6)	4.73	<0.001	1.7 (1,2.7)	1.7 (1,2.5)	1.65	0.098	2 (1.3,3)	1.9 (1.3,2.8)	3.03	0.002
HOMA-beta	61.6 (42.2,89.1)	59.9 (40.6,85.2)	2.18	0.029	54.3 (34.6,82.6)	53.7 (35.4,79.2)	0.61	0.545	65.5 (47.1,93)	66.7 (47,90.9)	−0.27	0.789
*Thyroid indices*												
FT3 (pmol/l)	4.9 (4.5,5.4)	5.1 (4.7,5.5)	−8.79	<0.001	5.2 (4.7,5.7)	5.3 (4.9,5.7)	−3.70	<0.001	4.8 (4.4,5.2)	4.9 (4.5,5.3)	−4.01	<0.001
FT4 (pmol/l)	15.9 (14.7,17.4)	15.8 (14.5,17.3)	2.29	0.022	16.2 (14.8,18)	16 (14.7,17.5)	2.50	0.013	15.8 (14.5,17.1)	15.6 (14.3,17)	2.58	0.010
TSH (mIU/l)	2.0 (1.5,2.7)	2.1 (1.5,2.8)	−2.24	0.025	1.8 (1.4,2.5)	1.9 (1.4,2.6)	−1.31	0.191	2.1 (1.6,2.8)	2.3 (1.7,3)	−4.38	<0.001
TPOAB (IU/ml)	13.8 (8.7,22.8)	12.5 (8.4,20.1)	4.23	<0.001	14.4 (9,23.5)	12.4 (8.2,19.1)	5.14	<0.001	13.3 (8.4,22.4)	12.7 (8.6,21.5)	0.851	0.395

SBP, systolic blood pressure; DBP, diastolic blood pressure; BMI, body mass index; WC, waist circumference; WHR, waist-to-hip ratio; TCH, total cholesterol; TG, triglyceride; HDL-C, high-density lipoprotein cholesterol; LDL-C, Low-density lipoprotein cholesterol; UA, uric acid; FPG, fasting plasma glucose; PPG, postprandial plasma glucose; HbA1c, Hemoglobin A1c; FINS, fasting plasma insulin; FT3, free triiodothyronine; FT4, free thyroxine; TSH, thyroid-stimulating hormone; TPOAB, thyroid peroxidase antibody.

**Table 2:** Stratified analysis of prevalence of thyroid nodules according to the different levels of metabolic syndrome components.

Parameters	Total subjects (*n* = 6798)		Males (*n* = 3289)		Females (*n* = 3509)	
	Thyroid nodules	Nonthyroid nodules	Thyroid nodules	Nonthyroid nodules	Thyroid nodules	Nonthyroid nodules
*Age (years)*						
<55	540 (24.1)	1700 (75.9)	194 (18.5)	854 (81.5)	346 (29.0)	846 (71.0)
55–65	912 (34.2)	1756 (65.8)	362 (27.2)	967 (72.8)	550 (41.1)	789 (58.9)
65–75	497 (39.4)	765 (60.6)	203 (32.3)	426 (67.7)	294 (46.5)	339 (53.6)
≥75	252 (41.0)	362 (59.0)	93 (34.1)	180 (65.9)	159 (46.6)	182 (53.4)
Chi-square	123.139		53.725		75.329	
*P* value	<0.001		<0.001		<0.001	
*P* for trend	<0.001		<0.001		<0.001	
*BMI (kg/m* ^*2*^)						
<25.0	1270 (32.8)	2602 (67.2)	465 (26.1)	1319 (73.9)	805 (38.6)	1283 (61.5)
25.0–30.0	755 (32.1)	1601 (68.0)	314 (25.3)	929 (74.7)	441 (39.6)	672 (60.4)
≥30.0	122 (32.5)	254 (67.6)	48 (28.9)	118 (71.1)	74 (35.2)	136 (64.8)
Chi-square	0.380		1.076		1.479	
*P* value	0.827		0.584		0.477	
*P* for trend	0.618		0.888		0.806	
*WHR*						
Q1	504 (31.1)	1115 (68.9)	187 (24.0)	593 (76.0)	317 (37.8)	522 (62.2)
Q2	515 (31.8)	1105 (68.2)	196 (24.7)	597 (75.3)	319 (38.6)	508 (61.4)
Q3	520 (32.1)	1099 (67.9)	203 (26.3)	568 (73.7)	317 (37.4)	531 (62.6)
Q4	544 (33.9)	1062 (66.1)	208 (26.8)	567 (73.2)	336 (40.4)	495 (59.6)
Chi-square	3.034		2.222		1.941	
*P* value	0.386		0.528		0.585	
*P* for trend	0.101		0.144		0.372	
*Waist circumference (cm)*						
<90 in men	812 (29.3)	1964 (70.8)	454 (25.1)	1356 (74.9)	358 (37.1)	608 (62.9)
<80 in women						
≥90 in men	1281 (34.6)	2425 (65.4)	345 (26.2)	973 (73.8)	936 (39.2)	1452 (60.8)
≥80 in women						
Chi-square	20.506		0.479		1.324	
*P* value	<0.001		0.489		0.250	
*HOMA-IR*						
<2.8	1603 (31.0)	3566 (69.0)	649 (24.9)	1954 (75.1)	954 (37.2)	1612 (62.8)
≥2.8	597 (37.0)	1016 (63.0)	203 (30.1)	472 (69.9)	394 (42.0)	544 (58.0)
Chi-square	20.194		7.366		6.758	
*P* value	<0.001		0.001		0.009	
*Glucose metabolic status*						
NGR, *n* (%)	472 (29.9)	1106 (70.1)	195 (26.1)	652 (73.9)	277 (33.3)	554 (66.7)
Prediabetes, *n* (%)	1094 (31.4)	2390 (68.6)	397 (24.1)	1248 (75.9)	697 (37.9)	1142 (62.1)
Diabetes, *n* (%)	526 (37.2)	889 (62.8)	208 (28.8)	515 (71.2)	318 (46.0)	374 (54.1)
Chi-square	20.773		5.746		25.896	
*P* value	<0.001		0.057		<0.001	
*P* for trend	<0.001		0.253		<0.001	
*UA (μmol/l)*						
≤420 in men	1856 (32.5)	3862 (67.5)	702 (26.6)	1933 (73.4)	1154 (37.4)	1929 (62.6)
≤360 in women						
>420 in men	341 (32.2)	719 (67.8)	150 (23.4)	492 (76.6)	191 (45.7)	227 (54.3)
>360 in women						
Chi-square	0.034		2.881		10.622	
*P* value	0.854		0.090		0.001	
*NAFLD*						
Without NAFLD	760 (32.2)	1599 (67.8)	254 (26.3)	713 (73.7)	506 (36.4)	886 (63.7)
NAFLD	535 (33.4)	1069 (66.7)	214 (26.0)	610 (74.0)	321 (41.2)	459 (58.9)
Chi-square	0.561		0.020		4.892	
*P* value	0.454		0.887		0.027	
*Metabolic syndrome*						
Without MetS	1168 (30.1)	2716 (69.9)	539 (25.6)	1566 (64.3)	629 (35.4)	1150 (64.6)
MetS	876 (35.3)	1605 (64.7)	249 (25.8)	716 (74.2)	627 (41.4)	889 (58.6)
Chi-square	19.041		0.014		12.500	
*P* value	<0.001		0.908		<0.001	

BMI, body mass index; WHR, waist-to-hip ratio; NGR, normal glucose regulation; UA, uric acid; NAFLD, nonalcoholic fatty liver disease; MetS, metabolic syndrome.

## Abbreviations

WHR: Waist-to-hip ratio
SBP: Systolic blood pressure
DBP: Diastolic blood pressure
BMI: Body mass index
WC: Waist circumference
HC: Hip circumference
NAFLD: Nonalcoholic fatty liver disease
TCH: Total cholesterol
TG: Triglyceride
HDL-C: High-density lipoprotein cholesterol
LDL-C: Low-density lipoprotein cholesterol
UA: Uric acid
FPG: Fasting plasma glucose
PPG: Postprandial plasma glucose
HbA1c: Hemoglobin A1c
FINS: Fasting plasma insulin
NGR: Normal glucose regulation
NAFLD: Nonalcoholic fatty liver disease
MetS: Metabolic syndrome
FT3: Free triiodothyronine
FT4: Free thyroxine
TSH: Thyroid-stimulating hormone
TPOAB: Thyroid peroxidase antibody.
